# ZrO_2_ coated Li_1.9_K_0.1_ZnTi_3_O_8_ as an anode material for high-performance lithium-ion batteries

**DOI:** 10.1039/d2ra05555d

**Published:** 2022-11-02

**Authors:** Jing Peng, Xianguang Zeng, Huafeng Zhu, Kui Xia, Jing Gong, Kaixin Huang

**Affiliations:** School of Materials Science and Engineering, Sichuan University of Science & Engineering Zigong 643000 China hnzxg1979@126.com; Material Corrosion and Protection Key Laboratory of Sichuan Province Zigong 643000 China; Langxingda Technology Co., Ltd Zigong 643000 China

## Abstract

The Li_1.9_K_0.1_ZnTi_3_O_8_@ZrO_2_ (1 wt%, 3 wt%, and 5 wt%) anode material was synthesized by doping Li_2_ZnTi_3_O_8_ with potassium and coating ZrO_2_, where the ZrO_2_ coating layer was prepared by citric acid and zirconium acetate, and the potassium source was KCl. When the added ZrO_2_ amount is 3%, the material has the most uniform size, reduced polarization, and reduced charge transfer resistance, and the specific capacity of LKZTO@ZrO_2_ (3 w%) was 361.5 mA h g^−1^ at 200 mA g^−1^ at the 100th cycle, which is higher than that of LKZTO, of 311.3 mA h g^−1^. The specific capacities of LKZTO@ZrO_2_ (3 w%) at 50, 100, 200, 500, and 1000 mA g^−1^ after 10 cycles were 424.9, 410.7, 394.1, 337.6 and 270.6 mA h g^−1^, indicating that LKZTO@ZrO_2_ (3 w%) has excellent electrochemical performance.

## Abstract 2

The Li_1.9_K_0.1_ZnTi_3_O_8_@ZrO_2_ (1 wt%, 3 wt%, and 5 wt%) anode material was successfully synthesized, where the ZrO_2_ coating layer was prepared by citric acid and zirconium acetate, and the potassium source was KCl.

## Graphical abstract

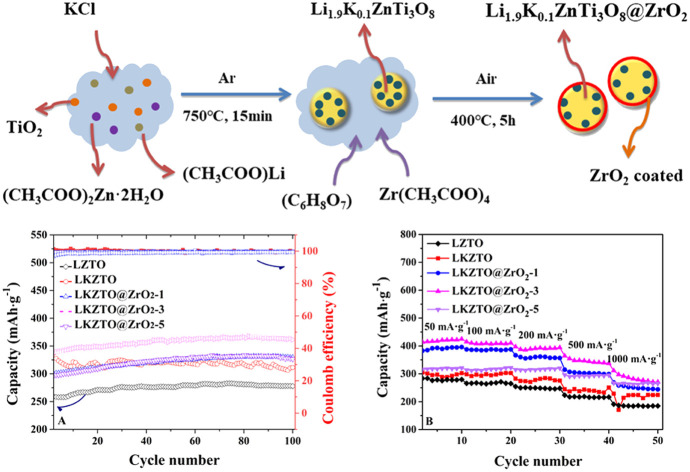

## 1 Introduction

Lithium-ion batteries (LIBs) are a new category of rechargeable batteries with a high specific capacity, high specific energy, long life, low cost, and high operating voltage, and have a wide range of application prospects. The physicochemical structure of the negative electrode active material has a decisive effect on the embedding and detachment of lithium ions, and is the carrier of lithium ions and electrons, which plays the role of energy storage and release, so the selection of anode materials is essential for good service life and charge/discharge performance of LIBs.^1–14^

Li_2_ZnTi_3_O_8_ (LZTO) with a cubic spinel structure is considered a promising material in view of its non-toxicity, low cost, and high theoretical capacity. Li^+^ and Zn^2+^ occupy the octahedral position 8c in an atomic ratio of 1 : 1, while Li^+^ and Ti^4+^ occupy the octahedral positions 4b and 12d respectively in an atomic ratio of 1 : 3, so that LZTOcan also be written as (Li_0.5_Zn_0.5_)tet (Li_0.5_Ti_1.5_)oct (where tet stands for the tetrahedral position, and oct represents the octahedral position). In particular, a unique three-dimensional meshwork structure made by LiO_6_ and TiO_6_ provides a channel for Li^+^ transport.^15,16^ Compared with lithium zinc titanate, silicon in the process of lithium-ion insertion/extraction will cause Si volume expansion of 100% to 300%, generate greater internal stress inside the material, and cause damage to the material structure; the electrode material falls off on the copper foil, and the SEI film on the silicon surface is constantly repeatedly formed-ruptured-formed, which jointly reduces the conductivity and cyclic stability of the electrode. However, the ionic and electronic conductivity of LZTO is relatively poor. To improve the low electrical conductivity of LZTO, the usual modification methods used are surface coating,^17–19^ ion doping,^20–25^ and structural nanosizing.^26^

Zeng *et al.*^22^ added Cr(NO_3_)_3_ to Li_2_ZnTi_3_O_8_ to achieve Cr^3+^ doping by the liquid phase process. The experimental results suggest that the discharge-specific capacities of Li_2_ZnTi_2.9_Cr_0.1_O_8_ were 156.7 and 107.5 mA h g^−1^ at 2 and 5 A g^−1^, respectively. Furthermore, even at 1 A g^−1^ the specific capacity remained at 162.2 mA h g^−1^ at the 200th cycle. The doping of Cr^3+^ improved the electrical conductivity of Li_2_ZnTi_2.9_Cr_0.1_O_8_, thus enhancing its electrochemical performance.

Li_2_ZnTi_3_O_8_@α-Fe_2_O_3_ were synthesized by Li *et al.*^18^ using a simple hydrothermal method. The Li_2_ZnTi_3_O_8_/α-Fe_2_O_3_ showed an irregular spherical morphology similar to that of Li_2_ZnTi_3_O_8_ and relatively small particle size compared to Li_2_ZnTi_3_O_8_. The charging capacity of Li_2_ZnTi_3_O_8_/α-Fe_2_O_3_ (5 wt%) was 184.8 mA h g^−1^, whereas the charging capacity of Li_2_ZnTi_3_O_8_ was 110.7 mA h g^−1^. The Li_2_ZnTi_3_O_8_/α-Fe_2_O_3_ has the benefits of a single component and exhibits new and attractive properties.

Tang *et al.*^19^ prepared Li_2_ZnTi_3_O_8_/La_2_O_3_ anode nanocomposites by a simple method. The high specific capacity of Li_2_ZnTi_3_O_8_/La_2_O_3_ was 188.6 mA h g^−1^ and maintained a high specific capacity of 147.7 mA h g^−1^ at the 100th cycle at 2.0 A g^−1^. Furthermore, Li_2_ZnTi_3_O_8_/La_2_O_3_ exhibited a retention rate of 42.7% after 1000 cycles at 2.0 A g^−1^, which was much higher than that of uncoated Li_2_ZnTi_3_O_8_. The superior lithium storage performance of Li_2_ZnTi_3_O_8_/La_2_O_3_ can be attributed to the stability of the protective layer, the small particle size, and the large surface area.

Zirconia is often used as a cladding material and ZrO_2_ is known to have three well-defined crystalline phases, namely cubic, tetragonal and monoclinic phases.^27^ In this paper, zirconia cladding materials were prepared using different ratios of citric acid and zirconium acetate as raw materials, lithium acetate anhydrous as lithium source, zinc acetate dihydrate as zinc source, titanium dioxide nanoparticles as titanium source, and potassium chloride as potassium source, and Li_1.9_K_0.1_ZnTi_3_O_8_ (LKZTO) was prepared by sol–gel combined with microwave sintering, and finally different amounts of ZrO_2_ were added to LKZTO. The LKZTO@ZrO_2_ anode material was obtained by coating with different amounts of ZrO_2_. The results showed that the electrochemical performance of the material prepared at 3% ZrO_2_ addition was superior.

## 2 Materials and methods

### 2.1 Preparation of LKZTO

LKZTO (0.02 mol) was prepared *via* the sol–gel reaction. First, TiO_2_ (99.8%, Maclin), (CH_3_COO)_2_Zn·2H_2_O (AR, Cologne Chemical), (CH_3_COO)Li (99.9%, Maclin), and KCl (AR, Cologne Chemical) were added to anhydrous ethanol, and the mixture was dried at 80 °C for 4 h. Then the white precursor was heated at 750 °C for 15 minutes in a microwave sintering furnace (in an Ar atmosphere) to prepared the LKZTO anode material, which named LKZTO. The preparation of LZTO is the same as the above scheme but does not contain KCl.

### 2.2 Preparation of LKZTO@ZrO_2_ composite

**Fig. 1:**
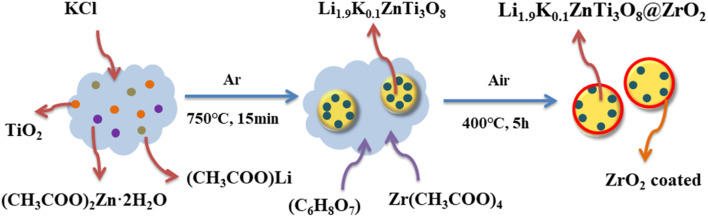
Preparation diagram of LKZTO@ZrO_2_.

For the preparation of LKZTO@ZrO_2_ composite, the prepared LKZTO was dispersed in deionized water for 30 minutes. After that, the citric acid (C_6_H_8_O_7_) and Zr(CH_3_COO)_4_ were dissolved in deionized water, respectively, and drop by drop into the LKZTO solution, the mixture was dried at 80 °C after sealed at 80 °C for 3 h. Then the white precursor was heated at 400 °C for 5 h in a muffle furnace. The ratios of ZrO_2_ in LKZTO are 1, 3, and 5 wt%, and the corresponding composites are named LKZTO@ZrO_2_-1, LKZTO@ZrO_2_-2, and LKZTO@ZrO_2_-3, respectively, as Fig. 1.

### 2.3 Materials characterization

The crystal structure of the synthesized material was obtained by X-ray diffraction (XRD, Brook AXS's D2 PHASER), and the range of records is 10–70° (2*θ*) with CuKα radiation. The micromorphology of the materials was observed by scanning electron microscope (SEM, TESCAN VEGA3) and high-resolution transmission electron microscope (HR-TEM, Talos F200X). The Brunauer Emmett Teller (BET) surface area tests were analyzed using an ASAP2460. Surface chemical composition was recorded by X-ray photoelectron spectroscopic (XPS, Escalab 250Xi) using a K Kα excitation source.

### 2.4 Electrochemical measurements

Electrochemical testing of anode materials was tested by a CR2032 button cell. Electrode materials were made up of active materials (80 wt%), Super P (10 wt%), and sodium carboxymethylcellulose (CMC) (10 wt%) (The loading capacity of the active material is about 1.0–1.5 mg, and the small loading amount will make the electrochemical performance of the material not be fully demonstrated, and the excessive loading amount will lead to material waste inside the material, resulting in a sharp decrease in the specific capacity of the material), then the slurry was spread on a copper foil and dried at 80 °C for 8 h in a vacuum oven. The CR 2032 button cells were assembled in an Ar-filled glove box. The constant current charge–discharge test was carried out on the LANHE CT2001A in the voltage range from 0.5 to 3.0 V (*vs.* Li/Li^+^).

Cyclic voltammetry (CV) and electrochemical impedance spectroscopy (EIS) tests were recorded by CHI660E. CV tests were recorded in the voltage range from 0.05 to 3.0 V at the scanning rate of 0.1 mV s^−1^, and EIS tests were measured at the frequency range of 10–10 kHz. All electrochemical properties of materials were measured at temperature.

## 3 Results and discussion

**Fig. 2:**
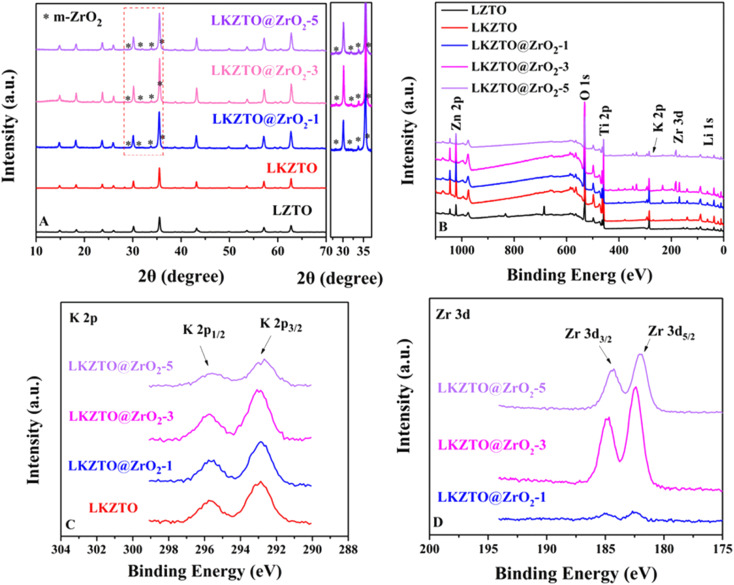
(A) XRD pattern, and (B) XPS pattern of LZTO, LKZTO, LKZTO@ZrO_2_-1, LKZTO@ZrO_2_-3 and LKZTO@ZrO_2_-5, (C) K 2p of LKZTO, LKZTO@ZrO_2_-1, LKZTO@ZrO_2_-3 and LKZTO@ZrO_2_-5, (D) Zr 3d of LKZTO@ZrO_2_-1, LKZTO@ZrO_2_-3 and LKZTO@ZrO_2_-5.

XRD patterns for LZTO, LKZTO, LKZTO@ZrO_2_-1, LKZTO@ZrO_2_-3 and LKZTO@ZrO_2_-5 materials are shown in Fig. 2(A). All samples have good crystallinity and belong to the cubic spinel structure, which indicates that a dose of K^+^ did not change the structure of the materials. It is worth noting that the 2*θ* degree of 28.3°, 31.5°, 33.9°, and 35.78° correspond to (1̄11), (111), (002) and (1̄02) planes of Monoclinic-ZrO_2_ (m-ZrO_2_),^28^ respectively.

To further study, the lattice parameters of five materials are expressed in Table 1. The results indicated that the increased lattice constant of LKZTO, which may be due to a slight increase in the lattice constant due to the entry of K^+^ into the crystal structure, as the radius of K^+^ (1.38 Å) is larger compared to Li^+^ (0.076 Å),^29^ and the lattice parameters only change slightly after ZrO_2_ coating, means ZrO_2_ only coated on the surface of the LKZTO and not change the cubic spinel structure, and The addition of ZrO_2_ makes the particle size of the material smaller, the particle size of the material is small, and the large surface tension makes the lattice distortion and the lattice parameters become smaller. It's showing that K^+^ doping and ZrO_2_ coating widens the transport channels for lithium ions and speeds up the rate of Li^+^ transport, further improving the electrochemical properties of the electrode material.^30^

**Table 1:** Lattice constants of LZTO, LKZTO, LKZTO@ZrO_2_-1, LKZTO@ZrO_2_-3 and LKZTO@ZrO_2_-5

Samples	Lattice parameters
	a (Å)
LKZTO@ZrO_2_-5	8.37454
LKZTO@ZrO_2_-3	8.37377
LKZTO@ZrO_2_-1	8.37629
LKZTO	8.37323
LZTO	8.36786

To prove the formation on the surface of the material, the X-ray photoelectron spectroscopy (XPS) spectrum of LZTO, LKZTO, and LKZTO@ZrO_2_ has shown in Fig. 2(B–D). The peaks of Li 1s, O 1s, Zn 2p, and Ti 2p appear in all samples while the peak of Zr 3d appears only in the LKZTO@ZrO_2_, which indicated that ZrO_2_ has only coated on the surface of LKZTO. In Fig. 2(C), two peaks at about 292.78 and 295.58 eV correspond well to K 2p_3/2_ and K 2p_1/2_,^31^ suggesting the presence of K^+^ in the LKZTO and LKZTO@ZrO_2_. The Zr 3d peak can be described as two main peaks at 182.4 and 184.8 eV corresponding to Zr 3d_5/2_ and Zr 3d_3/2_, representing the Zr^4+^ of ZrO_2_(ref. 32) (Fig. 2(D)), this is evidence that samples immersed only in Zr (CH_3_COO)_4_ have only surface Zr compared to non-immersion samples.

**Fig. 3:**
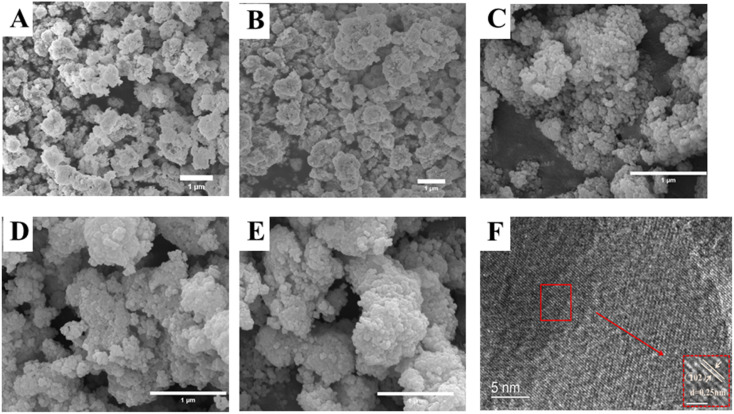
The SEM images of (A) LZTO, (B) LKZTO, (C) LKZTO@ZrO_2_-1, (D) LKZTO@ZrO_2_-3 and (E) LKZTO@ZrO_2_-5, HRTEM images (F) of LKZTO@ZrO_2_-3.

The SEM images of LZTO, LKZTO, and LKZTO@ZrO_2_ and the TEM and HRTEM images of LKZTO@ZrO_2_-3 are shown in Fig. 3. As seen in Fig. 3, the crystallinity of all samples is well, the particles are evenly distributed and the size is uniform. As can be seen from Fig. 3(A), the microscopic morphology of the material prepared under unmodified conditions with a diameter of about 200–300 nm; after K^+^ doping (Fig. 3(B)), the microscopic morphology with a diameter of about 200–300 nm. This shows that the diameter of the material does not change significantly after the doping of K^+^. Fig. 3(C–E) shows the SEM images of LKZTO@ZrO_2_-1, LKZTO@ZrO_2_-3 and LKZTO@ZrO_2_-5. From Fig. 3(C–E), it can be seen that when the zirconia addition is 1%, the size distribution of LKZTO@ZrO_2_-1 material is not homogeneous; when the zirconia content continues to increase to 5%, the material shows aggregation, which may be caused by too much zirconia addition; when the addition is 3%, LKZTO@ZrO_2_-3 were homogeneous in size, around 60–70 nm, compared to the size of 200–300 nm for LKZTO, the addition of zirconia reduced the size of the material to a great extent.

The material LKZTO@ZrO_2_-3 with a ZrO_2_ addition of 3% was selected and analyzed by transmission electron microscopy. It is obvious from the TEM images that there is a layer of cladding material on the surface of the material, and the cladding layer was placed under a high-resolution transmission microscope for observation to obtain Fig. 3(F). The lattice striations of the material are evident in Fig. 3(F), indicating its good crystallinity. Calculation of the crystal plane spacing in the selected areas shows that the crystal plane spacing of the cladding layer is all 0.25 nm, which corresponds to the (1̄02) crystal plane of monoclinic zirconia and is consistent with the XRD results, the peak with m-ZrO_2_ is detected by XRD, and the (1̄02) crystal plane with m-ZrO_2_ is corresponded to TEM, indicating that LKZTO@ZrO_2_ was successfully prepared.

**Fig. 4:**
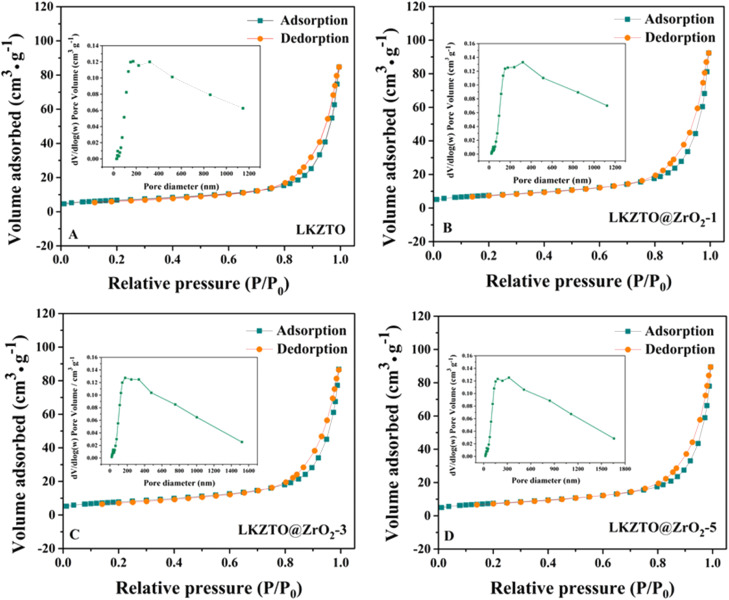
The nitrogen adsorption–desorption isotherms of (A) LKZTO, (B) LKZTO@ZrO_2_-1, (C) LKZTO@ZrO_2_-3, (D) LKZTO@ZrO_2_-5 and corresponding pore size distributions (inset).

The specific surface area and pore diameter of LKZTO and LKZTO@ZrO_2_ has displayed in Fig. 4(A–D). The specific surface area of LKZTO, LKZTO@ZrO_2_-1, LKZTO@ZrO_2_-3, and LKZTO@ZrO_2_-5 is 24.0615, 26.8465, 27.6906, and 26.6244 m^2^ g^−1^ (relative error of ± 6% and the error was small which had little influence on the results), and it is clear that the surface area of LKZTO@ZrO_2_-3 is largest. The specific surface area of the material increased, which shortens the transmission distance of Li^+^, and reduces the charge transfer resistance and the degree of polarization. Moreover, the larger area makes the transmission of Li^+^ more efficient and prevents the side reaction between the electrode material and the electrolyte. Meanwhile, it can stabilize the structure and improve the material's chemical properties.

**Fig. 5:**
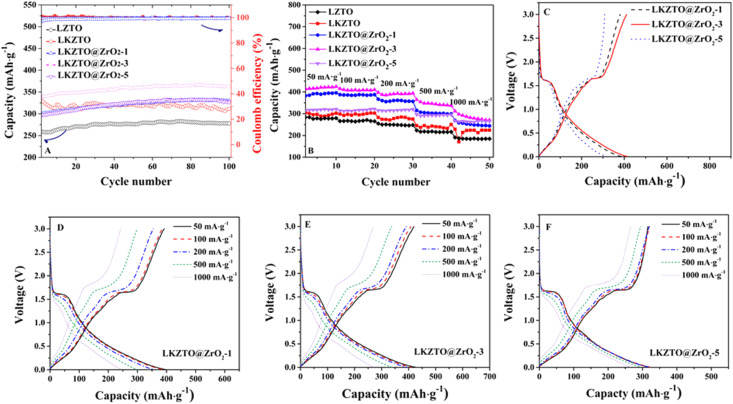
(A) Cycle performance and (B) rate performance of LZTO, LKZTO, LKZTO@ZrO_2_-1, LKZTO@ZrO_2_-3, and LKZTO@ZrO_2_-5, (C) third charge and discharge curve of LKZTO@ZrO_2_-1, LKZTO@ZrO_2_-3 and LKZTO@ZrO_2_-5, (D)–(F) first charge and discharge curve of the different current density of LKZTO@ZrO_2_-1, LKZTO@ZrO_2_-3, and LKZTO@ZrO_2_-5, respectively.

The cycling performance of LZTO, LKZTO, and LKZTO@ZrO_2_ was characterized at 200 mA g^−1^ (Fig. 5(A)) between 0.5–3.0 V. The capacity of LZTO, LKZTO, LKZTO@ZrO_2_-1, LKZTO@ZrO_2_-3, and LKZTO@ZrO_2_-5 is 259.1, 337.1, 300.1, 338.1 and 296.4 mA h g^−1^ after two cycles. At the 100th cycle, the capacity of 277.9, 311.3, 329.5, 361.5, and 326.5 mA h g^−1^ for LZTO, LKZTO, LKZTO@ZrO_2_-1, LKZTO@ZrO_2_-3, and LKZTO@ZrO_2_-5, respectively. The capacity of LKZTO@ZrO_2_-3 has increased means the polarization degree of the material is reduced, and the charge transfer resistance is reduced. Moreover, the transport of Li^+^ is more efficient, and the electrochemical performance of the material is improved.

The rate performance is an important way to evaluate the excellent electrochemical performance of a material. The material is cycled from a small current to a high current cycle, which shows whether the structure of the material will collapse due to too much current. The rate properties of LZTO, LKZTO, and LKZTO@ZrO_2_ are compared in Fig. 5(B). At 50 mA g^−1^, the capacity of LZTO, LKZTO, LKZTO@ZrO_2_-1, LKZTO@ZrO_2_-3, and LKZTO@ZrO_2_-5 are 283.5, 304.9, 382.8, 413.9, and 316.3 mA h g^−1^ at the second cycle. The specific capacities of LKZTO@ZrO_2_-3 after 10 cycles of 50, 100, 200, 500, and 1000 mA g^−1^ each were 424.9, 410.7, 394.1, 337.6, and 270.6 mA h g^−1^, while the specific capacities of LKZTO@ZrO_2_-1 after 10 cycles of 50, 100, 200, 500 and 1000 mA g^−1^ were 396, 387.6, 356.8, 299.2 and 244.2 mA h g^−1^, and the specific capacities of LKZTO@ZrO_2_-5 after 10 cycles of 50, 100, 200, 500 and 1000 mA g^−1^ were 321.1, 322, 320.2, 295, and 265.4 mA h g^−1^, while the specific capacities of LKZTO after 10 cycles of each 50, 100, 200, 500, and 1000 mA g^−1^ were 302.3, 302.7, 275.6, 250.66 and 224.4 mA h g^−1^, and the comparison shows that LKZTO@ZrO_2_-3 has the best multiplicative performance. The rate performance of the material was greatly improved after the appropriate amount of K^+^ doping and ZrO_2_ coating, probably due to:^33^ (1) K^+^ doping reduces charge transfer resistance and enhances conductivity, giving LKZTO good electrochemical properties; (2) the low dose of potassium doping broadens the Li^+^ transport channel, increasing the rate of Li^+^ transport; (3) the coating of ZrO_2_ can reduce the charge transfer resistance and enhance the electrical conductivity, which gives LKZTO@ZrO_2_-3 a good electrochemical performance; (4) the appropriate amount of ZrO_2_ coating makes the polarization lower and the Li^+^ transport efficiency higher; (5) hinder the side reaction between the material and electrolyte, the structure of the material is more stable, and the electrochemical performance of the material is improved.

Fig. 5(C) shows the charge/discharge curves of LKZTO@ZrO_2_-1, LKZTO@ZrO_2_-3, and LKZTO@ZrO_2_-5 at the third cycle at 50 mA g^−1^, (D) – (F) shows the charge/discharge curves of LKZTO@ZrO_2_-1, LKZTO@ZrO_2_-3, and LKZTO@ZrO_2_-5 at different current densities. From Fig. 5(C), it can be seen that the discharge-specific capacity at the third cycle of LKZTO@ZrO_2_-3 at 50 mA g^−1^ is 415.5 mA h g^−1^, and the third cycle, while the discharge-specific capacities of LKZTO@ZrO_2_-1 and LKZTO@ZrO_2_-5 were 385.1 mA h g^−1^ and 317 mA h g^−1^, respectively, and the comparison shows that when the addition of ZrO_2_ is 3%, the initial discharge specific capacity of LKZTO@ZrO_2_-3 is better than that of LKZTO@ZrO_2_-1 and LKZTO@ZrO_2_-5.

Fig. 5(D) and (E) show that the discharge-specific capacity of the material has a very obvious decreasing trend with the increase of current density, while graph (F) shows that its discharge-specific capacity decreases slowly with the increase of current density, which may be because when the addition of ZrO_2_ is 1%, although the specific capacity of the material increases, the outer layer of the material has limited ZrO_2_ coating, and the electrode material will have some side reactions generated with the electrolyte to consume Li^+^, so its specific capacity also decreases faster with the decrease of current density. When the addition amount is 3% and 5%, the ZrO_2_ in the outer layer of the material prevents the material from reacting with the electrolyte, which makes the material more stable and the specific capacity decreases more slowly. Therefore, when the addition amount of ZrO_2_ is 3%, the specific capacity can have a higher increase and also maintain a better structure under the increasing current without a rapid decrease in specific capacity, which indicated the rate capability of LKZTO@ZrO_2_-3 is better than that of LKZTO@ZrO_2_-1 and LKZTO@ZrO_2_-5.

The insertion of lithium ions into LKZTO involves the following processes: (1) the lithium ions dissolved in the electrolyte diffuse onto the surface of LKZTO; (2) a charge–transfer reaction occurs at the interface between LKZTO and the electrolyte, accepting both electrons from the collector and lithium ions from the electrolyte; (3) Lithium ions diffuse into the LKZTO. After coating ZrO_2_ could affect the processes of the charge–transfer reaction occurs at the interface between LKZTO and the electrolyte, and the lithium ions diffuse from the electrolyte to the surface of the ZrO_2_ coating and diffuse to the internal structure of the material through the porous structure. This will allow lithium ions to diffuse into the material more quickly through the porous structure, allowing more efficient electron transport. Obviously, a smaller particle sizes will shorten the transmission distance of Li^+^, and reduces the charge transfer resistance and polarization. Further, ZrO_2_ coating can hinder the direct contact between the electrode material and the electrolyte, reducing the probability of side reactions occurring, thereby maintaining the structural stability of the material while inhibiting the formation of SEI.

**Fig. 6:**
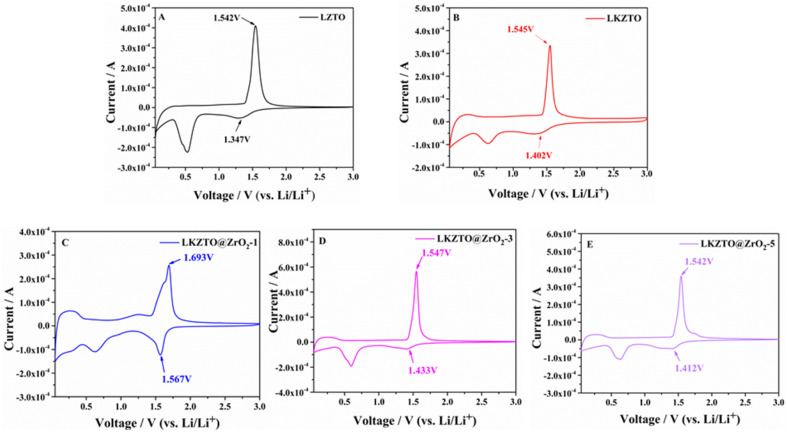
CV curves of (A) LZTO, (B) LKZTO, (C) LKZTO@ZrO_2_-1, (D) LKZTO@ZrO_2_-3 and (E) LKZTO@ZrO_2_-5.

Fig. 6 shows the cyclic voltammetry curves of LZTO, LKZTO, LKZTO@ZrO_2_-1, LKZTO@ZrO_2_-3, and LKZTO@ZrO_2_-5, recorded at a scan rate of 0.1 mV s^−1^ with potentials ranging is 0.05–3 V. The peak anode potential (φPa), the peak cathode potential (φPc) and the difference between the peak anode and cathode (ΔφP) are shown in Table 2. LZTO, LKZTO, LKZTO@ZrO_2_-1, LKZTO@ZrO_2_-3, and LKZTO@ZrO_2_-5 all showed a pair of redox peaks in the range of 1–2 V, which corresponded to the Ti^4+^/Ti^3+^ redox process,^34,35^ and the shapes of the curves did not change significantly, which indicated that K^+^ doping and ZrO_2_ coating does not change the electrochemical process of LZTO.^36,37^ From Fig. 6, it can also be found that there is a reduction peak around 0.3–0.6 V, which may correspond to the multi-bit storage of Ti^4+.^^37,38^

**Table 2:** Electrochemical parameters (potential difference between anodic and cathodic peaks from the CV)

Sample	φPa (v)	φPc (V)	ΔφP (mV)
LKZTO@ZrO_2_-5	1.542	1.412	130
LKZTO@ZrO_2_-3	1.547	1.433	114
LKZTO@ZrO_2_-1	1.693	1.567	126
LKZTO	1.545	1.402	143
LZTO	1.542	1.347	195

The potential differences between the anodic and cathodic peaks of LZTO, LKZTO, LKZTO@ZrO_2_-1, LKZTO@ZrO_2_-3, and LKZTO@ZrO_2_-5 were 195, 143, 126, 114 and 130 mV, respectively, as shown in Table 2, which indicates that among the five materials, LKZTO@ZrO_2_-3 has the smallest redox potential difference of 114 mV. It is well known that the potential difference between the anodic and cathodic peaks (ΔφP) can reflect the strength of the reversibility of an electrochemical process.^39,40^ Therefore, LKZTO@ZrO_2_-3 has smaller polarization than LKZTO@ZrO_2_-1 and LKZTO@ZrO_2_-5, the transport efficiency of Li^+^ is increased, and the materials exhibit superior electrochemical performance properties, which are consistent with the test results of cycling performance and rate performance. Therefore, coating ZrO_2_ is a better way to improve the electrochemical performance of the electrode material LZTO.

**Fig. 7:**
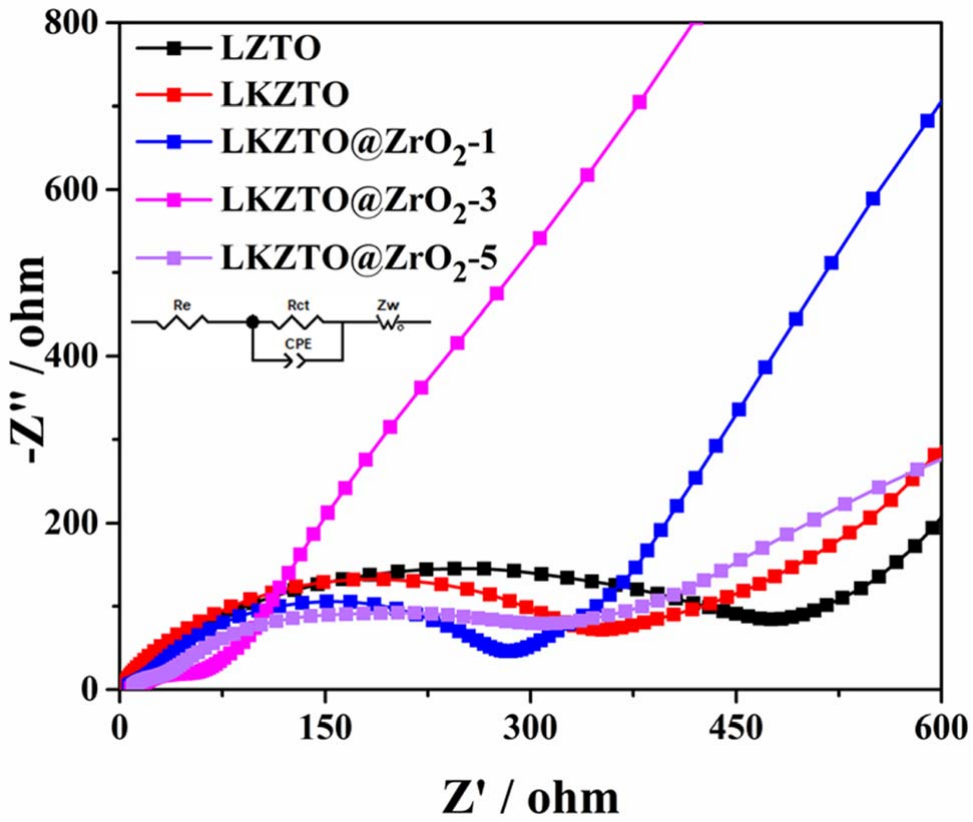
EIS curves of LZTO, LKZTO, LKZTO@ZrO_2_-1, LKZTO@ZrO_2_-3 and LKZTO@ZrO_2_-5.

To better understand the electrochemical reaction behavior of LZTO, LKZTO, LKZTO@ZrO_2_-1, LKZTO@ZrO_2_-3, and LKZTO@ZrO_2_-5, EIS tests were performed on the five materials. The EIS spectra (Fig. 7.) all consist of a semicircle and a diagonal line, where the intercept of the semicircle in the high-frequency region with the *X*-axis represents the ohmic resistance, *i.e.* the contact resistance between the electrolyte and the electrode and between the electrode and the diaphragm; the semicircle represents the charge transfer resistance within the electrode and the diagonal line represents the Warburg impedance caused by the diffusion of Li^+^ in the active material.^17,41^ The corresponding equivalent circuit is shown in the inset of Fig. 7, where re represents the contact resistance in the liquid phase, *R*_ct_ represents the charge transfer resistance, CPE is a constant, and *Z*_w_ is the Warburg resistance.^42–44^ It is obvious from Fig. 7 that the charge transfer resistance of LKZTO@ZrO_2_-3 is the lowest, and after fitting, the charge transfer resistance of LZTO, LKZTO, LKZTO@ZrO_2_-1, LKZTO@ZrO_2_-3, and LKZTO@ZrO_2_-5 were 476.3, 334.5, 278.9, 68.51 and 333.7 Ω, respectively, from which it can be seen that there is a significant decrease in the charge transfer resistance of the materials after the addition of ZrO_2_, which indicates that the cladding of ZrO_2_ is an effective method to enhance the electrical conductivity and reduce the resistance. In addition, from the low-frequency region, the Li^+^ diffusion rate of LKZTO@ZrO_2_-3 is slightly higher than that of LKZTO@ZrO_2_-1 and LKZTO@ZrO_2_-5.

## 4 Conclusions

The LKZTO@ZrO_2_ (1 wt%, 3 wt%, and 5 wt%) anode material was synthesized by doping LZTOwith potassium and coating ZrO_2_, which the ZrO_2_ coating layer was prepared by citric acid and zirconium acetate, and the potassium source was KCl. From TEM and XRD can prove that the LKZTO@ZrO_2_ was successfully prepared, from SEM can observe that the distribution of the material is uniform, the size is uniform, and the size of the material is reduced after coating ZrO_2_, which is conducive to shortening the transport path of lithium ions. The specific capacities of LKZTO@ZrO_2_-3 after 10 cycles of 50, 100, 200, 500, and 1000 mA g^−1^ each were 424.9, 410.7, 394.1, 337.6, and 270.6 mA h g^−1^. After 100 cycles at 200 mA g^−1^, the capacity of 361.5 mA h g^−1^ of LKZTO@ZrO2-3 demonstrates that LKZTO@ZrO_2_ is a great anode material for high-performance lithium-ion batteries.

## Author contributions

Xianguang Zeng and Jing Peng: contributed conception and design of the study. Huafeng ZHU and Kaixin Huang: organized the database. Jing Peng, Jing Gong, and Kui Xia: wrote the first draft of the manuscript. Xianguang Zeng: revised the whole manuscript.

## Conflicts of interest

The authors declare that the research was conducted in the absence of any commercial or financial relationships that could be construed as a potential conflict of interest.

## Supplementary Material
